# Diffuse large B cell non-Hodgkin lymphoma complicated with jejunal stricture and perforation

**DOI:** 10.1016/j.ijscr.2024.110375

**Published:** 2024-09-30

**Authors:** Prajjwol Luitel, Ishwor Thapaliya, Sujan Paudel, Simanta Khanal, Ratan Shah, Pawan Sapkota Upadhya

**Affiliations:** a Maharajgunj Medical Campus, Institute of Medicine, Tribhuvan University, Kathmandu, Nepal; b Department of General Surgery, B.P. Koirala Institute of Health Sciences, Dharan, Nepal

**Keywords:** Diffuse large B-cell lymphoma, Perforation, Stricture, Surgery, Chemotherapy

## Abstract

**Introduction:**

DLBCL with gastrointestinal involvement is a relatively rare form of extra-nodal lymphoma, and complications such as spontaneous perforation and jejunal stricture in this disease are even rarer.

**Case presentation:**

A 45-year-old male presented with abdominal pain and vomiting. Diagnosis revealed a jejunal stricture with perforation, necessitating resection and anastomosis. Histopathology performed after surgery confirmed DLBCL, and the patient was referred for chemotherapy.

**Discussion:**

DLBCL with gastrointestinal involvement is uncommon and often manifests with nonspecific symptoms, leading to diagnostic delays. Treatment includes addressing both lymphoma and associated complications, with surgical intervention reserved for emergencies.

**Conclusion:**

Bowel perforation and jejunal stricture in non-Hodgkin's lymphoma are serious complications requiring prompt treatment to improve outcomes and reduce mortality.

## Highlights

• DLBCL with gastrointestinal involvement is rare and can lead to serious complications like perforation and stricture.
• Nonspecific symptoms often cause diagnostic delays in gastrointestinal DLBCL.
• Chemotherapy remains the primary treatment, but surgery is essential for managing complications.
• Close monitoring and comprehensive care are vital for improving outcomes in DLBCL with gastrointestinal involvement.

## 1 Introduction

Primary gastrointestinal lymphoma, although rare, is the most common form of extra-nodal lymphoma and typically presents as a non-Hodgkin type [1]. Among non-Hodgkin lymphomas, diffuse large B-cell lymphoma (DLBCL) is the predominant subtype among older population, accounting for approximately 30 % of cases annually [2]. DLBCL commonly affects the stomach (50–60 %) and the small bowel (30–40 %) [1]. The complications such as perforation and bleeding occur in less than 2 % of cases [3,4], with spontaneous bowel perforation in absence of chemotherapy being even rarer [5].

Diagnosis in patients with localized abdominal pain and nonspecific gastrointestinal symptoms relies heavily on imaging studies and pathological tissue review [6]. Following the SCARE guidelines [7], we present a case of DLBCL that presented with abdominal pain and vomiting, necessitating surgical intervention due to acute abdomen.

## 2 Case presentation

A 45-year-old South Asian male presented to the emergency department with a history of severe generalized abdominal pain and multiple episodes of vomiting over 24 h. There was a history of altered bowel habits, intermittent abdominal pain, and per rectal bleeding over the past year. He was a non-smoker and had no significant history of alcohol consumption or medications. There was no significant family history of gastrointestinal or hematological malignancy.

On arrival, his vital signs included a temperature of 37.5 °C, heart rate of 96 bpm, blood pressure of 110/70 mmHg, and respiratory rate of 18/min. Generalized abdominal tenderness were present.

Laboratory studies showed White Blood cell (WBC) count of 14,000/μL, hemoglobin of 12.5 g/dL, normal renal function, and blood glucose of 110 mg/dL. The abdominal X-ray revealed free gas under the right hemidiaphragm, suggesting a gastrointestinal perforation.

**Fig. 1:**
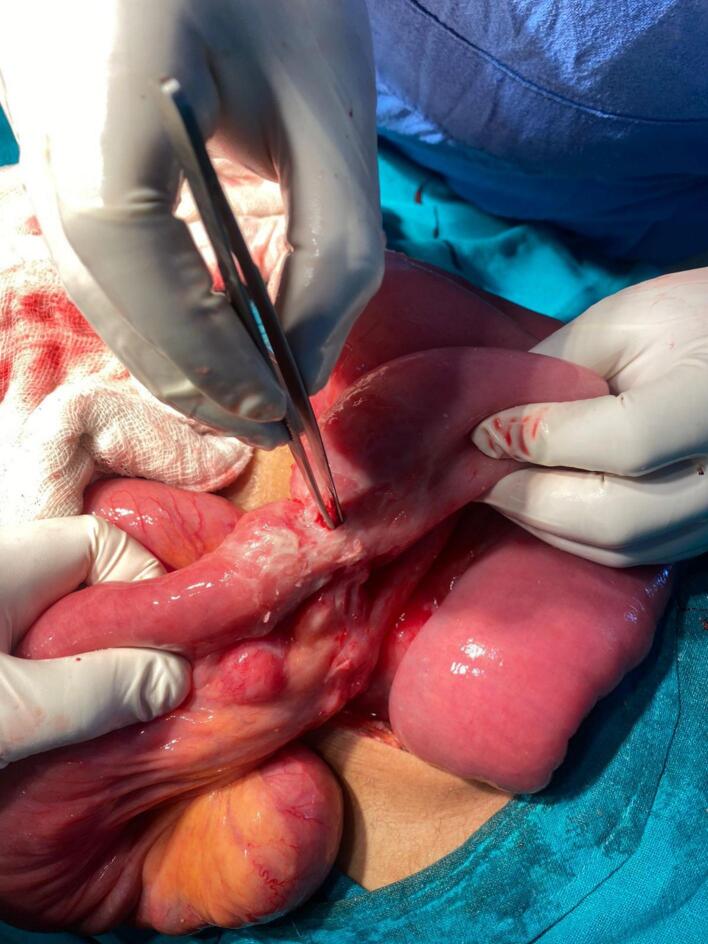
Intraoperative image showing perforation on jejunum and stricture.

Given the clinical findings, a provisional diagnosis of hollow viscous perforation peritonitis was made. He was kept Nil per oral, received intravenous fluids, antibiotics (Ceftriaxone and Metronidazole) and morphine for analgesia. Exploratory laparotomy under general anesthesia was performed urgently by team headed by senior surgeon. Intraoperative findings revealed gross contamination and a stricture at the jejunum, located 70 cm from the duodenojejunal flexure, with a 0.5 × 0.5 cm perforation at the site of the stricture (Fig. 1).

There was no other organ involvement. The proximal jejunal segment was grossly dilated, while the distal segment collapsed. Additionally, multiple enlarged mesenteric lymph nodes, majority measuring greater than 3 cm, were observed. 10 cm proximal segment and 5 cm of distal of jejunum along with stricture was resected and end to end anastomosis was performed. The enlarged mesenteric lymph nodes were excised for biopsy. A drain was placed in the left pelvic cavity, with the tip positioned near the site of the anastomosis for potential leaks.

Postoperatively, he was admitted in the Intensive care unit. He was started on a clear liquid diet, which was gradually advanced to a soft diet by the fourth postoperative day. Pelvic drain was removed on the sixth postoperative day. His postoperative course was uneventful. By the time of discharge, the patient was tolerating regular diet and had normal bowel and bladder habits.

The pathological examination of the stricture revealed diffuse proliferation of medium to large sized neoplastic lymphoid cells extending up to the *peri* muscular fibrous tissue. Proximal and distal cut margins were free of tumor. Lymph node revealed diffuse proliferation of large lymphoid cells with infiltration into pericapsular fat. The findings were suggestive of DLBCL. Immunohistochemistry revealed positivity for CD15, CD30, CD79a, MUM1, and Ki-67. It was negative for CD10, CD136, Bcl-6, Bcl-2. Bone marrow aspiration revealed normocellular marrow. A Computed tomography (CT) revealed multiple prominent mesenteric and para-aortic nodes, largest measuring 30 × 27 mm. Retropharyngeal, masticator, parotid, and carotid spaces were normal, with a notable left side level IV node measuring approximately 10 mm. On further follow-up, the patient recovered well. There was no surgical site infection. He was referred to tertiary care center for further staging and chemotherapy to address the DLBCL.

## 3 Discussion

DLBCL with gastrointestinal involvement is relatively rare, comprising 10 %–30 % of extra-nodal lymphomas [2]. It typically occurs in individuals in their sixth decade and is slightly more prevalent in males [8]. Studies have shown that the small intestine, particularly the ileum (60–65 %) and jejunum (20–25 %), is the second most commonly affected site in non-Hodgkin's lymphoma, followed by the duodenum (6–8 %) and other sites (8–9 %) [9]. Small-bowel involvement in NHL can manifest as solitary or multiple nodules, circumferential wall thickening with or without aneurysmal dilatation, and direct extension from mesenteric nodes [10].

Patients with primary gastrointestinal lymphoma usually present with nonspecific symptoms such as abdominal pain, anorexia, and weight loss [1]. B symptoms (fever, night sweats, and weight loss) are less common compared to nodal lymphomas, contributing to diagnostic delays [5]. The disease may mimic tuberculosis (TB), particularly in HIV-positive patients and regions where TB is endemic, underscoring the importance of excluding this condition [9].

The exact cause of DLBCL with gastrointestinal involvement is unclear, however genetic abnormalities, immune system dysregulation, chronic inflammation, and viral infections are thought to play a role in progression of disease [2]. Risk factors implicated in the pathogenesis of gastrointestinal lymphoma include HIV, Coeliac disease, Epstein-Barr virus, Hepatitis B, inflammatory bowel disease, and *Helicobacter pylori* [9].

Dawson et al. identified five key criteria for diagnosing primary malignant lymphoma of the intestinal tract: no palpable superficial lymphadenopathy, absence of enlarged mediastinal lymph nodes on chest radiographs, normal total and differential white blood cell counts, notable bowel lesions observed during laparotomy, and no tumors present in the liver or spleen [11]. The patient had a jejunal stricture and perforation without infiltration of other organs. Since the present case fulfilled all the criteria mentioned, it was diagnosed as primary DLBCL.

Gastrointestinal perforation often occurs at sites affected by lymphoma cells [12]. Perforation in DLBCL differs between chemotherapy-treated and untreated cases. Chemotherapy-induced perforation results from weakened tissue due to rapid tumor necrosis and lysis [5]. Spontaneous perforation occurs in two patterns: from ulceration and tumor necrosis extending to the subserosa, or from an ulcer with thin connective tissue and no tumor involvement [3]. Stricture in NHL-involved bowel loops may result from mucositis and inflammation, where cytokines like tumor necrosis factor and interleukin-1 contribute to increased intestinal smooth muscle cell proliferation [10].

Treatment selection should be based on tumor location, clinical stage, pathological pattern, and the presence or absence of complications [5]. Chemotherapy is the primary treatment for all types of NHL. However, DLBCL with complications such as perforation or stricture requires surgical resection [9]. Management of the perforation of the GI lymphoma involves addressing both lymphoma and associated complications, with critical emphasis on intraoperative decision-making [3]. Although surgery is no longer the primary treatment for this condition, jejunectomy with reconstruction, along with adjuvant medical therapy are recommended when complications such as benign strictures arise due to the increased risk of disease progression without surgical resection of the original tumor [4,5]. Our patient initially underwent emergency surgery for perforation and jejunal stricture. Subsequently, due to mesenteric and para-aortic lymph node involvement, he was referred to higher center for chemotherapy.

Female gender and B-cell phenotype predict better prognosis, while radical surgery improves outcomes; however, perforation and strictures worsen prognosis [6]. Close monitoring, comprehensive supportive care, advanced personalized treatment strategies and research into biomarker are essential for managing DLBCL with gastrointestinal involvement [2].

## 4 Conclusion

Bowel perforation with jejunal stricture in non-Hodgkin's lymphoma is a serious complication requiring prompt diagnosis and treatment to reduce high mortality rates. Further research is essential to clarify surgical indications, pathological findings, and understand the clinical impact of spontaneous perforation and stricture in primary intestinal lymphoma.

## Consent

Written informed consent was obtained from the patient for publication and any accompanying images. A copy of the written consent is available for review by the Editor-in-Chief of this journal on request.

## Declaration

All the authors declare that the information provided here is accurate to the best of our knowledge.

## Ethical approval

Since this is a case report, the Institutional Review Board of the Institutional Review Committee, Institute of Medicine, has waived the requirement for ethical approval.

## Funding

No funding received.

## Author's contribution

P.L., I.T., and S.P. formulated the original manuscript. P.L., I.T., S.P., S.K., and P.S. reviewed and edited the manuscript. S.K., R.S., and P.S. supervised the case. All the authors reviewed and approved the final version of the manuscript.

## Guarantor

Prajjwol Luitel

## Declaration of competing interest

All the authors declare that they have no conflict of interest.
